# Quinoxalines against *Leishmania amazonensis*: SAR study, proposition of a new derivative, QSAR prediction, synthesis, and biological evaluation

**DOI:** 10.1038/s41598-023-45436-1

**Published:** 2023-10-24

**Authors:** Anna Carolina Silva de Jesus Passaes, Juliana Arantes Dantas, Fernanda Landim Lopes, Diego Pereira Sangi, Magaly Girão Albuquerque, Celso Vataru Nakamura, Julliane Yoneda

**Affiliations:** 1 Departamento de Química, Instituto de Ciência Exatas, Universidade Federal Fluminense, Volta Redonda, 27213-145 Brazil; 2 Programa de Pós-Graduação em Química, Universidade Federal de São Carlos, São Carlos, 13565-905 Brazil; 3 Programa de Pós-Graduação em Química, Departamento de Química Orgânica, Instituto de Química, Universidade Federal do Rio de Janeiro, Rio de Janeiro, 21941-909 Brazil; 4 Programa de Pós-Graduação em Ciências Farmacêuticas, Universidade Estadual de Maringá, Maringá, 87020-900 Brazil

**Keywords:** Organic chemistry, Theoretical chemistry

## Abstract

Neglected tropical diseases, such as leishmaniasis, lead to serious limitations to the affected societies. In this work, a structure–activity relationship (SAR) study was developed with a series of quinoxaline derivatives, active against the promastigote forms of *Leishmania amazonensis*. As a result, a new quinoxaline derivative was designed and synthesized. In addition, a quantitative structure–activity relationship (QSAR) model was obtained [pIC_50_ = − 1.51 − 0.96 (E_HOMO_) + 0.02 (PSA); N = 17, R^2^ = 0.980, R^2^_Adj_ = 0.977, *s* = 0.103, and LOO-cv-R^2^ (Q^2^) = 0.971]. The activity of the new synthesized compound was estimated (pIC_50_ = 5.88) and compared with the experimental result (pIC_50_ = 5.70), which allowed to evaluate the good predictive capacity of the model.

## Introduction

According to the World Health Organization (WHO), the neglected tropical diseases (NTDs) are a diverse group of 20 conditions that are mainly prevalent in the tropical and subtropical regions of the world, such as Latin America, Africa, and Asia, predominantly in developing countries. The NTDs are considered endemic in low-income populations, as they affect vulnerable people, who in most cases have limited access to clean and potable water, and poor hygiene and sanitation conditions^1^.

Among the 20 NTDs, tuberculosis, Chagas disease, leprosy, malaria, dengue, schistosomiasis, and leishmaniasis are included in the Brazilian National Agenda of Priorities in Health Research^2^. Although they have been present on our planet for thousands of years, they continue without being eradicated, and impose serious limitations on the affected societies, leading to a panorama of illness, suffering, disability, and death, with serious social, economic, and psychological consequences, affecting more than 1 billion people worldwide, according to the WHO data^3^.

In January 2021, WHO launched its new roadmap for tackling NTDs from 2021 to 2030. Targets include achieving prevention, control, elimination, and eradication of a diverse set of diseases until 2030. The goal includes a 90% decrease in the number of people in need of treatment, that at least one hundred countries eliminate at least one neglected disease present in their nation, and a 75% reduction in years of life lost due to disability caused by these diseases^3^.

The treatments available for the NTDs are very limited and insufficient, in addition to presenting a series of problems, such as low efficacy, high toxicity, and the emergence of resistant strains^4^.

Leishmaniasis are a set of diseases caused by protozoan of the Leishmania genus and the Trypanosomatidae family, and they are transmitted to humans by the bite of infected female phlebotomine sandflies. Leishmaniasis can occur in three different clinical forms: (i) visceral leishmaniasis (VL), which is generally fatal without treatment; (ii) cutaneous leishmaniasis (CL) that causes skin ulcers; and (iii) mucocutaneous leishmaniasis (MCL), affecting nose, mouth, and throat. The WHO estimates that 700,000–1 million new cases occur each year worldwide^5^.

The antileishmanial treatment is performed, as a first choice, with pentavalent antimonial agents, such as sodium stibogluconate. Meanwhile, amphotericin B, pentamidine, miltefosine, and paromomycin have also been used with varying results. However, the current drugs have undesirable side effects and important toxicities. For example, amphotericin and pentavalent antimonial drugs cause high nephrotoxicity and cardiac effects, respectively, in addition to being administered intravenously, which sometimes hinders the patient’s adherence to the treatment, resulting in therapeutic failures and favoring the parasite’s resistance to the drugs^5,6^.

**Figure 1:**
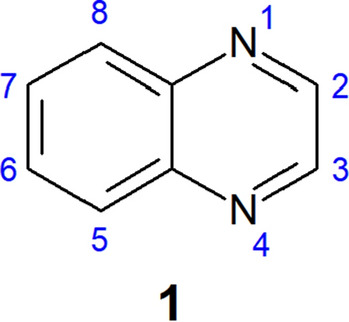
Chemical structure and atom numbering of the quinoxaline nucleus (**1**).

Some N-heterocyclic nucleus are considered privileged scaffolds due to their broad spectrum of biological activity highlighted in the literature^7^. Privileged scaffolds generally exhibit physicochemical properties that allow a single class of molecules to provide potent and selective ligands for different biological targets^8^. The *N*-heterocycle quinoxaline (1,4-naphthyridine) (Fig. 1) is considered a privileged scaffold.

Quinoxaline derivatives represent a class of biologically active compounds, showing anti-inflammatory, anticancer, antibacterial, antimicrobial, antifungal, antiviral, and antileishmanial activities, among others^8–12^. The quinoxaline heteroaromatic scaffold is found in more than 30 drugs available in the DrugBank (DB, https://go.drugbank.com/), including approved, nutraceuticals, investigational or experimental ones, such as brimonidine (DB00484), chlorsulfaquinoxaline (DB12921), erdafitinib (DB12147), rabeximod (DB05772), riboflavin (DB00140), and varenicline (DB01273).

On the search of new drug leads, there is a need for efficient and robust procedures that can be used to screen chemical databases against molecules with known activities. To this end, quantitative structure–activity relationships (QSAR) studies provide a mean for rationalizing the relationship between chemical structure and its biological action towards the development of new drug candidates^13^.

Cherkasov et al.^14^ have described several QSAR studies where computational and medicinal chemists worked together to discover novel molecules with unique biological activities.

In this context, this work aims to evaluate by an in silico approach which physicochemical properties of the quinoxaline derivatives (**2a–2i**, **3a–3i**, and **4a–4d**) (Table 1)^11^ contribute to their in vitro inhibitory activity against the promastigote forms of *Leishmania amazonensis*, to propose and to synthesize a new potential antileishmanial agent, and to build a QSAR model able to predict its activity.

**Table 1:** Chemical structures of the quinoxaline derivatives (**2a–2i**, **3a–3i**, and **4a–4d**) and the corresponding in vitro inhibitory activities (IC_50_, μM) against the promastigote forms of *Leishmania amazonensis*^11^.


#	R^1^	R^2^	R^3^	R^4^	IC_50_ (µM)	pIC_50_ (M)
**2a**	H	OMe	NMe_2_	SMe	42.8	4.37
**2b**	H	OMe	NH(n-Bu)	SMe	35.2	4.45
**2c**	H	OMe	NH(cyclohexyl)	SMe	29.8	4.53
**2d**	OMe	H	NH(isopentyl)	SMe	27.1	4.57
**2e**	Br	H	NH(n-Bu)	SMe	25.2	4.60
**2f**	H	OMe	NH(isobutyl)	SMe	27.6	4.56
**2g**	Cl	H	NH(n-Bu)	SMe	24.4	4.61
**2h**	OMe	H	NH(isobutyl)	SMe	26.9	4.57
**2i**	OMe	H	NH(n-Bu)	SMe	30.2	4.52
***3a***	*H*	*OMe*	*NH(n-Bu)*	*SO*_2_*Me*	*2.5*	*5.60*
***3b***	*H*	*OMe*	*NH(cyclohexyl)*	*SO*_2_*Me*	*2.9*	*5.54*
***3c***	*Br*	*H*	*NH(n-Bu)*	*SO*_2_*Me*	*1.6*	*5.80*
***3d***	*H*	*OMe*	*NH(isobutyl)*	*SO*_2_*Me*	*2.6*	*5.59*
***3e***	*Cl*	*H*	*NH(n-Bu)*	*SO*_2_*Me*	*1.4*	*5.85*
***3f***	*Cl*	*H*	*NH(cyclohexyl)*	*SO*_2_*Me*	*2.2*	*5.66*
***3g***	*H*	*H*	*NH(n-Bu)*	*SO*_2_*Me*	*2.9*	*5.54*
***3h***	*Br*	*H*	*NH(EtOH)*	*SO*_2_*Me*	*0.8*	*6.10*
***3i***	*Br*	*H*	*Cl*	*SO*_2_*Me*	*0.2*	*6.70*
**4a**	Cl	Cl	Ph	Ph	5.3	5.28
**4b**	H	H	4-OMe-Ph	4-OMe-Ph	30.0	4.52
**4c**	H	H	4-Me-Ph	Ph	8.9	5.05
**4d**	H	H	4-OMe-Ph	Ph	12.8	4.89

## Methods

### Computational chemistry

The three-dimensional (3D) structures of the quinoxaline derivatives (**2a–2i**, **3a–3i**, and **4a–4d**) (Table 1) were constructed using the Spartan’10 software (Wavefunction, Inc.)^15^. Each structure was submitted to a full geometry optimization step by a molecular mechanics model, using the Merck molecular force field (MMFF), available in the Spartan software. Then, each optimized structure was submitted to the default systematic conformational analysis at Spartan, using the same molecular mechanics force field. The lowest-energy conformer for each quinoxaline derivative was submitted to a full geometry optimization (energy minimization) step by a semi-empirical model, using the Austin Method 1 (AM1) Hamiltonian at Spartan. Then, each optimized conformer was submitted to a single-point energy calculation by a density functional theory (DFT) model, using the B3LYP hybrid DFT method at Spartan, considering the 6-311 +  + G(*d*,*p*) basis set. For each energy minimized DFT structure, the following thirteen physicochemical properties were obtained: total energy (E_T_, au), energy of the highest occupied molecular orbital (E_HOMO_, eV), energy of the lowest unoccupied molecular orbital (E_LUMO_, eV), HOMO–LUMO energy gap (GAP, eV), dipole moment (μ, Debye), base-10 logarithm of the partition coefficient (LogP), surface area (SA, Å^2^), molecular volume (MV, Å^3^), molecular weight (MW, amu), polarizability (P, 10^−30^ m^3^), number of hydrogen bond donors (HBD), number of hydrogen bond acceptors (HBA), and polar surface area (PSA, Å^2^).

A linear cross-correlation matrix was constructed with the calculated thirteen physicochemical properties as a criterion to exclude at least one from the two highly correlated pair of properties and generate a subset of properties to be used in the QSAR equations construction. Therefore, the calculated values of a set of selected properties were set as the independent variables (X) used to calculate the QSAR equations along with the values of the dependent variable (Y), i.e., the biological activity values, which were converted from IC_50_ (μM) (Table 1) to the corresponding pIC_50_ (M) values, before the QSAR equations generation. Then, the QSAR equations were obtained by multiple linear regression (MLR) analysis, using the Microsoft Excel^®^ program (Microsoft Inc.).

In addition, using the OSIRIS Property Explorer server^16^, the toxicity risks of the quinoxaline derivatives were evaluated in silico and fragment-based drug-likeness score was calculated in the same server.

### Organic synthesis

**Figure 2:**
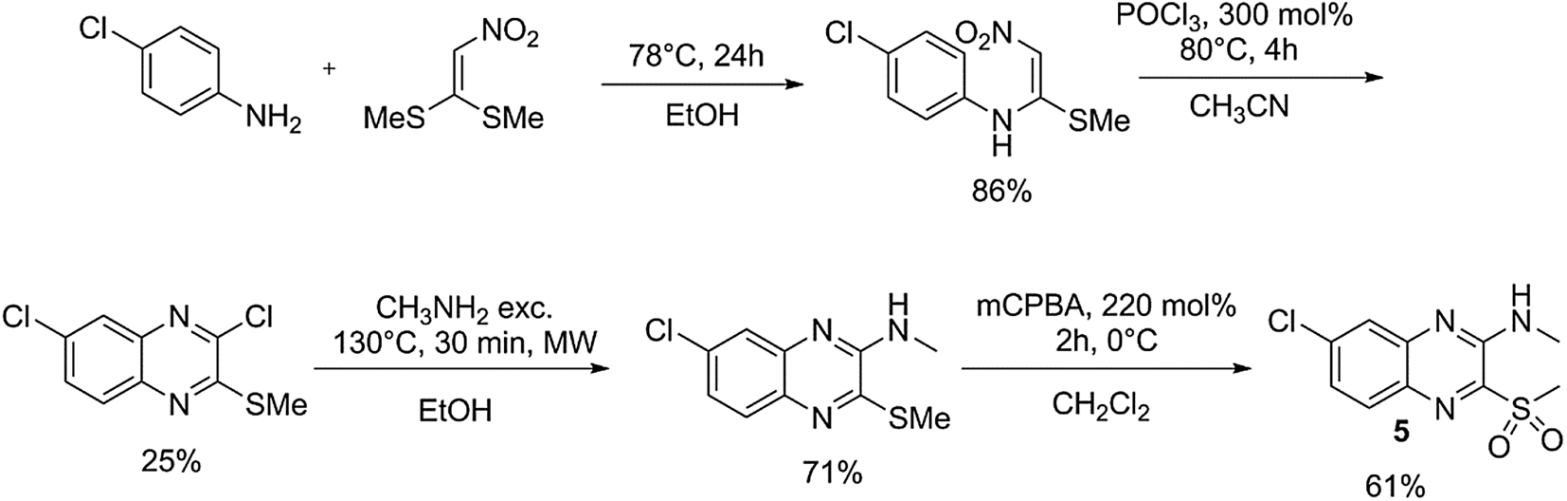
Synthetic route of 7-chloro-*N*-methyl-3-(methylsulfonyl)quinoxalin-2-amine (**5**).

The proposed quinoxaline derivative (5) was synthesized following the route used by Cogo et al.^11^ in the synthesis of 2-amino-3-sulfonylquinoxalines. This synthetic route consists in four steps: (i) vinylic substitution of 1,1-bis(methylsulfanyl)-2-nitroethene using 4-chloroaniline as nucleophile and ethanol as solvent to obtain the 4-chloro-*N*-(1-methylsulfanyl-2-nitroethenyl)aniline intermediate; (ii) cyclization of the intermediate with phosphoryl chloride (POCl_3_) to produce the pyrazine ring of the quinoxaline nucleus of the 3,6-dichloro-2-methylsulfanylquinoxaline intermediate, using acetonitrile as solvent; (iii) microwave assisted nucleophilic substitution, using ethanol as solvent to install the methylamino substituent in the 2-position of the quinoxaline nucleus; and (iv) oxidation of the methylsulfanyl group with 3-chloroperbenzoic acid (mCPBA) to obtain the sulfone (5) in dichloromethane as solvent (Fig. 2).

The ^1^H NMR spectra of all intermediates and final product were obtained by using a Bruker ARX-400 equipment (400 MHz).

### In vitro growth inhibition assay

Promastigote (1 × 10^6^ cells/mL) cultures were inoculated in a 24-well plate in the absence or presence of different concentrations of the quinoxaline derivatives (0.1 and 100 μM). The inhibitory activity was evaluated after 72 h. The cell density for each concentration was determined by counting in a hemocytometer (Improved Double Neubauer). The concentration that inhibited cell growth in 50% (IC_50_) was determined by nonlinear regression analysis^11^.

## Results and discussion

### SAR analysis of the quinoxaline derivatives and design of a new derivative

Many descriptors reflect simple molecular properties give an insight referent to physicochemical nature of the observed biological activity^17^.

Table 2 shows the physicochemical descriptor values calculated at the DFT(B3LYP)/6-311 +  + G(*d*,*p*) level of theory for the quinoxaline derivatives (**2a–2i**, **3a–3i**, and **4a–4d**). All the most active quinoline derivatives (IC_50_ < 3 μM, i.e., pIC_50_ from 5.54 to 6.70 M, compounds **3a-3i**, see Table 1) presented the number of hydrogen bond acceptors (HBA) ranging from 5 to 7, the polar surface area (PSA) values ranging from 46 to 74 Å^2^, and the LUMO energy (E_LUMO_) values more negative than − 2.5 eV. In addition, the LogP values range from 1.6 to 3.5, and the HOMO energy (E_HOMO_) values are more negative than − 5.9 eV.

**Table 2:** Physicochemical descriptors calculated at the DFT(B3LYP)/6-311 +  + G(d,p) level of theory for the quinoxaline derivatives (**2a–2i**, **3a–3i**, and **4a–4d**) using the Spartan’10 software.

#	E_T_	E_HOMO_	E_LUMO_	GAP	μ	LogP	SA	MV	MW	P	HBD	HBA	PSA
**2a**	− 1104.17	− 5.80	− 1.84	3.96	1.88	2.65	257.98	253.34	249.34	60.98	0	4	22.26
**2b**	− 1182.83	− 5.59	− 1.69	3.90	2.35	3.51	319.31	289.61	277.39	63.94	0	4	30.50
**2c**	− 1260.28	− 5.56	− 1.65	3.91	2.28	3.82	336.25	313.11	303.43	65.85	0	4	30.07
**2d**	− 1222.16	− 5.60	− 1.58	4.02	2.08	4.03	334.48	306.94	291.42	65.32	0	4	28.70
**2e**	− 3641.82	− 6.01	− 1.97	4.04	3.75	4.47	309.23	280.13	326.26	63.14	0	0	23.30
**2f**	− 1182.83	− 5.59	− 1.67	3.92	2.34	3.14	314.10	289.20	277.39	62.91	0	4	28.27
**2g**	− 1527.90	− 5.99	− 1.94	4.05	3.60	4.19	304.46	275.52	281.81	62.77	0	3	23.29
**2h**	− 1182.83	− 5.63	− 1.62	4.01	1.79	3.14	314.10	289.20	277.39	63.88	0	4	28.15
**2i**	− 1182.83	− 5.61	− 1.59	4.02	2.05	3.51	319.28	289.58	277.39	63.91	0	4	29.85
***3a***	* − 1333.23*	* − 6.01*	* − 2.54*	*3.47*	*4.19*	*2.48*	*333.49*	*302.54*	*309.39*	*65.09*	*1*	*7*	*60.56*
***3b***	* − 1410.68*	* − 5.95*	* − 2.52*	*3.43*	*3.88*	*2.78*	*350.48*	*326.03*	*335.43*	*67.01*	*1*	*7*	*59.98*
***3c***	* − 3792.22*	* − 6.49*	* − 2.79*	*3.70*	*2.93*	*3.43*	*323.38*	*293.04*	*358.26*	*64.27*	*1*	*6*	*53.34*
***3d***	* − 1333.24*	* − 6.03*	* − 2.54*	*3.49*	*4.36*	*2.46*	*331.71*	*302.28*	*309.39*	*65.07*	*1*	*7*	*60.01*
***3e***	* − 1678.30*	* − 6.48*	* − 2.76*	*3.72*	*2.99*	*3.16*	*318.58*	*288.42*	*313.81*	*63.89*	*1*	*6*	*53.32*
***3f***	* − 1755.75*	* − 6.41*	* − 2.74*	*3.67*	*2.84*	*3.47*	*335.55*	*311.91*	*339.85*	*65.81*	*1*	*6*	*52.73*
***3g***	* − 1218.68*	* − 6.33*	* − 2.62*	*3.71*	*3.94*	*2.60*	*303.53*	*275.14*	*279.36*	*62.82*	*1*	*6*	*53.38*
***3h***	* − 3788.81*	* − 6.62*	* − 2.84*	*3.78*	*1.83*	*1.67*	*292.75*	*263.92*	*346.21*	*61.89*	*2*	*7*	*73.77*
***3i***	* − 4039.16*	* − 7.48*	* − 3.22*	*4.26*	*5.52*	*2.88*	*243.84*	*220.52*	*321.58*	*58.26*	*0*	*5*	*46.79*
**4a**	− 1799.52	− 6.65	− 2.60	4.05	3.36	6.52	341.44	332.33	351.24	67.37	0	2	14.52
**4b**	− 1109.39	− 5.94	− 2.16	3.78	2.35	5.15	371.49	360.45	342.40	69.72	0	4	28.79
**4c**	− 919.61	− 6.37	− 2.24	4.13	1.14	5.89	332.14	324.08	296.37	66.68	0	2	14.52
**4d**	− 994.84	− 6.11	− 3.22	2.89	1.86	5.28	341.21	333.16	312.37	67.48	0	3	21.65

Data for the most active compounds (**3a–3i**) (IC_50_ < 3 μM, see Table 1) are highlighted in italic.

E_T_, total energy (au); E_HOMO_, energy (eV) of the highest occupied molecular orbital; E_LUMO_, energy (eV) of the lowest unoccupied molecular orbital; GAP, HOMO–LUMO gap energy (eV); μ, dipole moment (Debye); LogP, base-10 logarithm of the partition coefficient; SA, surface area (Å^2^); MV, molecular volume (Å^3^); MW, molecular weight (amu); P, polarizability (10^−30^ m^3^), HBD, number of hydrogen bond donors (NH + OH), HBA, number of hydrogen bond acceptors (N + O); PSA, polar surface area (Å^2^).

Unfortunately, the fragment-based drug-likeness values predicted by the OSIRIS server for these compounds are negative like most of the Fluka chemicals that have negative values, whereas 80% of the commercial drugs have a positive drug-likeness value. Toxicity was also predicted by the OSIRIS, and compounds **3g** and **4a–d** showed alerts of mutagenic risks. On the other hand, **3d** showed the highest drug-score value (0.63). The drug-score index combines drug-likeness, cLogP (lipophilicity), LogS (water solubility), MW, and toxicity risks in one value used to predict the compound's overall potential as a drug.

Lipinski’s rule-of-five^18^ proposes that poor absorption or cell permeability of a drug occurs when its chemical structure fulfils more than one of the following criteria: the molecular weight (MW) is greater than 500 Daltons; the calculated LogP is greater than 5; the number of hydrogen bond donors (NH + OH) are more than 5; and the number of hydrogen bond acceptors (N + O) are more than 10. According to the Veber’s rule^19^, for good oral availability, the PSA value must be less than or equal to 140 Å^2^. The physicochemical properties calculated for the studied compounds fit these parameters, except the LogP values for **4a–d** (Table 2).

In order to improve these parameters, structural modifications on the studied compounds were proposed to design an antileishmanial agent with higher chances to become a drug.

Cogo and co-workers^11^ noticed that hydrogen replacement at R1 position (Table 1) by halogen elements (Cl or Br) increases the activity, and substitution at R2 position (Table 1) did not show great interference on the activity. The methylsulfonyl group is present in all the most active compounds studied in this work (Table 1) and literature data also indicates that it is one of the main groups at 3-position of quinoxaline derivatives, which are responsible for the observed activity against *Trypanosoma cruzi* and *Leishmania amazonensis*^11^.

**Figure 3:**
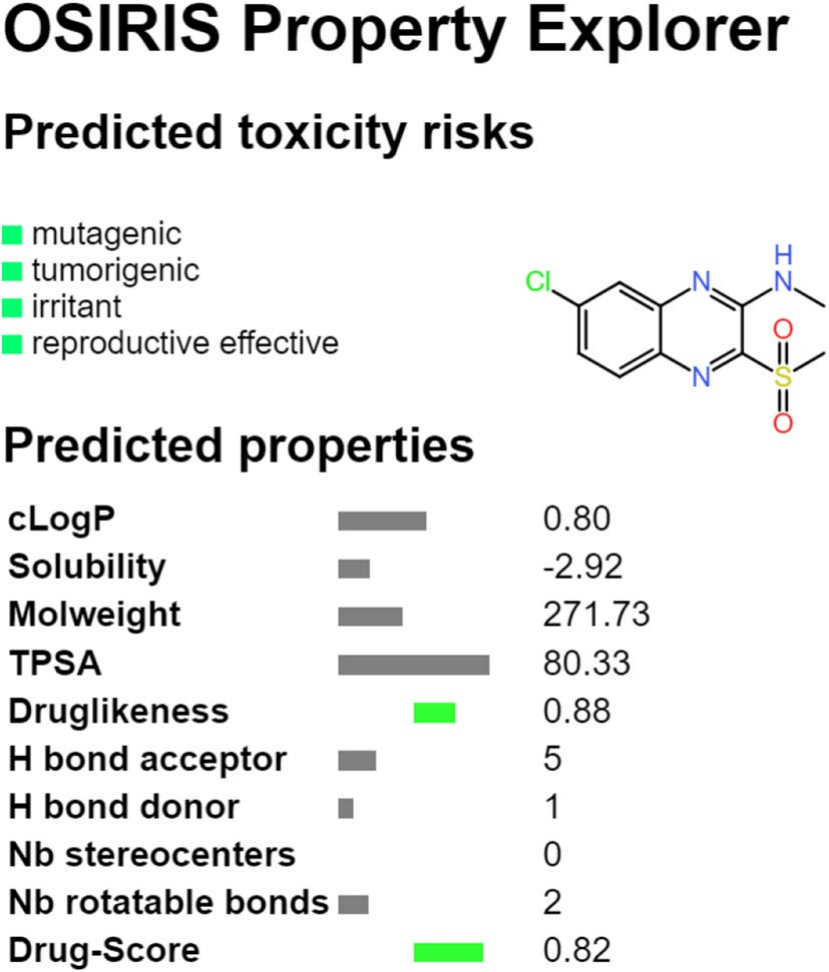
Toxicity risks and physicochemical properties predicted by the OSIRIS Property Explorer server for compound (**5**), 7-chloro-N-methyl-3-(methylsulfonyl)quinoxalin-2-amine, proposed as a potential antileishmanial agent.

Based on this SAR analysis, several structural modifications were proposed and their synthetic viability as well as the OSIRIS Property Explorer’s risk alerts were evaluated. After that, some of the designed compounds were selected for structural optimization and calculation of the corresponding physicochemical properties. Considering the properties related to the biological activity, compound **5** was proposed as a potential antileishmanial agent (Fig. 3).

It fulfilled all the requirements, presenting the physicochemical descriptors according to the most active compounds of the studied series: LUMO energy of − 2.79 eV, five H-bond acceptors, polar surface area of 53.18 Å^2^, LogP equal to 1.74, and HOMO energy of −  6.52 eV.

Besides, according to the OSIRIS Property Explorer server, compound 5 (Fig. 3) seems to have low toxicity risks (green color) and the drug-likeness and drug-score indexes were improved to 0.88 and 0.82, respectively, when compared to the other compounds of the series. It is also important to mention that compound 5 follows Lipinski’s rule-of-five and Veber’s rule related to PSA range of drug candidates.

### QSAR model construction and validation

A QSAR model was built to predict the activity value of compound 5. Firstly, the degree of correlation between all pairs among the thirteen descriptors (Table 1) was verified by constructing a cross-correlation matrix. After removing multicollinear descriptors, seven of them were selected (E_T_, E_HOMO_, E_LUMO_, dipole moment, LogP, MW, and PSA), and equations that describe the dependency relationship between the independent (X, properties or descriptors) and dependent (Y, biological activity) variables were obtained based on Hansh and Unger’s work^20^, who suggest that, in a selection of independent variables, for each independent variable included in the QSAR model, there must be no more than five observations (i.e., compounds), thus avoiding chance correlation^21^.

Therefore, the calculated values of those seven descriptors (Table 1) were set as the independent (X) variables used to calculate the QSAR equations along with the values of the dependent (Y) variable (i.e., biological activities), which were converted from IC_50_ (μM) (Table 1) to the corresponding pIC_50_ (M) values, before the QSAR equations generation.

Among the main methods used in the selection of the independent variables in QSAR, we applied the systematic search method, which consists in combining the available independent variables to build and analyze all possible linear regression equations. In the QSAR method, compounds are generally divided into training set and test set, compounds from the training set are used in the construction of QSAR equations and compounds from the test set are used in validation. Since there are 22 compounds (Table 1) and that part of them (~ 20% from the total number of compounds) should be removed from the model as a test group, we used a maximum of three independent variables to be included in each equation, considering N = 18 for the training set and N = 4 for the test set (namely, compounds **2i**, **3g**, **3h**, and **4d**).

The systematic search generated 63 regression equations: seven equations with one independent variable, 20 equations with two independent variables, and 34 equations with three independent variables. Tables 3, 4 and 5 list the previously selected independent variables included in the linear equations and the following statistical parameters of each equation calculated by the Microsoft Office Excel^®^ program (Microsoft Inc.): correlation coefficient (R), coefficient of determination (R^2^), adjusted coefficient of determination (R^2^_Adj_), standard error (s) and F-test.

**Table 3:** Statistical data for the seven QSAR equations with one term (N = 18 and *p* = 0.05), generated by systematic combination of the seven theoretical physicochemical descriptors.

Eq.	E_T_	E_HOMO_	E_LUMO_	μ	LogP	MW	PSA	R	R^2^	R^2^_Adj_	s	F
1	X							0.541	0.293	0.249	0.579	6.624
2		**X**						**0.997**	**0.995**	**0.936**	**0.390**	**3119.914**
3			X					0.947	0.897	0.890	0.222	138.659
4				X				0.687	0.472	0.439	0.501	14.315
5					X			0.266	0.071	0.013	0.664	1.220
6						X		0.561	0.315	0.272	0.570	7.359
7							X	0.702	0.493	0.461	0.490	15.567

**Table 4:** Statistical data for the 20 QSAR equations with two terms (N = 18 and *p* = 0.05) generated by systematic combination of seven theoretical physicochemical descriptors.

Eq.	E_T_	E_HOMO_	E_LUMO_	μ	LogP	MW	PSA	R	R^2^	R^2^_Adj_	s	F
8	X	X						0.843	0.711	0.672	0.383	18.441
9	X		X					0.948	0.898	0.885	0.227	66.260
10	X			X				0.710	0.504	0.438	0.501	7.624
11	X				X			0.590	0.348	0.261	0.575	4.003
12	X					X		0.635	0.404	0.324	0.549	5.079
13	X						X	0.808	0.653	0.606	0.419	14.100
14		X	X					0.947	0.897	0.883	0.229	65.065
15		X		X				0.882	0.777	0.747	0.336	26.142
16		X			X			0.928	0.861	0.843	0.265	46.504
17		X				X		0.852	0.726	0.689	0.373	19.826
18		**X**					**X**	**0.985**	**0.971**	**0.967**	**0.122**	**247.15**1
19			X	X				0.951	0.904	0.892	0.220	70.911
20			X		X			0.964	0.929	0.920	0.189	98.758
21			X			X		0.954	0.910	0.898	0.213	76.049
22			X				X	0.959	0.920	0.909	0.201	86.098
23				X	X			0.688	0.474	0.404	0.516	6.753
24				X		X		0.753	0.566	0.509	0.469	9.794
25				X			X	0.785	0.616	0.565	0.441	12.053
26					X	X		0.725	0.526	0.463	0.490	8.326
27					X		X	0.769	0.592	0.538	0.454	10.887
28						X	X	0.771	0.594	0.540	0.453	10.992

**Table 5:** Statistical data for the 34 QSAR equations with three terms (N = 18 and *p* = 0.05) generated by systematic combination of seven theoretical physicochemical descriptors.

Eq.	E_T_	E_HOMO_	E_LUMO_	μ	LogP	MW	PSA	R	R^2^	R^2^_Adj_	s	F
29	X	X	X					0.948	0.898	0.877	0.235	41.278
30	X	X		X				0.887	0.787	0.742	0.340	17.288
31	X	X			X			0.932	0.869	0.841	0.266	30.989
32	X	X				X		0.853	0.727	0.669	0.385	12.440
33	**X**	**X**					**X**	**0.986**	**0.97**3	**0.967**	**0.121**	**167.267**
34	X		X	X				0.951	0.904	0.884	0.228	44.193
35	X		X		X			0.965	0.931	0.916	0.193	62.979
36	X		X			X		0.957	0.915	0.897	0.214	50.399
37	X		X				X	0.962	0.925	0.908	0.202	57.251
38	X			X	X			0.714	0.509	0.404	0.516	4.842
39	X			X		X		0.756	0.572	0.480	0.482	6.225
40	X			X			X	0.822	0.676	0.607	0.419	9.741
41	X				X	X		0.749	0.560	0.466	0.488	5.948
42	X				X		X	0.850	0.723	0.663	0.388	12.157
43	X					X	X	0.821	0.675	0.605	0.420	9.675
44		X	X	X				0.951	0.905	0.884	0.228	44.228
45		X	X		X			0.970	0.941	0.929	0.178	74.858
46		X	X			X		0.954	0.910	0.891	0.221	47.389
47		X	X				X	0.986	0.972	0.966	0.124	160.655
48		X		X	X			0.931	0.866	0.838	0.269	30.254
49		X		X		X		0.886	0.784	0.738	0.342	16.954
50		X		X			X	0.985	0.971	0.965	0.126	155.383
51		X			X	X		0.958	0.918	0.900	0.211	52.002
52		X			X		X	0.985	0.971	0.964	0.126	153.837
53		X				X	X	0.986	0.972	0.966	0.124	160.256
54			X	X	X			0.964	0.930	0.915	0.195	61.996
55			X	X		X		0.957	0.917	0.899	0.213	51.360
56			X	X			X	0.960	0.922	0.906	0.205	55.310
57			X		X	X		0.965	0.930	0.915	0.194	62.278
58			X		X		X	0.964	0.929	0.914	0.196	61.500
59			X			X	X	0.965	0.931	0.917	0.193	63.229
60				X	X	X		0.787	0.619	0.537	0.455	7.585
61				X	X		X	0.833	0.694	0.628	0.407	10.581
62				X		X	X	0.820	0.672	0.602	0.422	9.579
63					X	X	X	0.775	0.601	0.515	0.465	7.019

Comparing the best equations (highlighted in bold on Tables 3, 4 and 5) of the three groups containing one (Eqs. 1–7), two (Eqs. 8–28) and three (Eqs. 29–63) theoretical physicochemical descriptors (independent variables or terms), i.e., Eq. 2 (pIC_50_ = − 0.84 (E_HOMO_), R^2^_Adj_ = 0.936), Eq. 18 (pIC_50_ = − 1.58–0.97 (E_HOMO_) + 0.02 (PSA), R^2^_Adj_ = 0.967), and Eq. 33 (pIC_50_ = − 1.85 + 4.31 × 10^−5^ (E_T_) − 1.02 (E_HOMO_) + 0.02 (PSA), R^2^_Adj_ = 0.967), respectively, the E_HOMO_ term is present in all of them. However, Eq. 2 should be excluded because it has the lowest R^2^_Adj_ value (a normalized R^2^ value, used to compare equations containing a different number of terms).

Therefore, considering only Eqs. 18 and 33, we can observe that the inclusion of the E_T_ term in Eq. 33 does not alter the R^2^_Adj_ value, which makes these two equations to be equivalent. Nevertheless, since the parsimony principle advises the choice of the simplest model, Eq. 18 was used to calculate the antileishmanial activity value for the 18 quinoxaline derivatives (Table 6), using as descriptors the E_HOMO_ and PSA independent variables (Table 4).

**Table 6:** Observed (experimental) and calculated (Eq. 18) pIC_50_ (M) values, residuals (pIC_50_ (observed)–pIC_50_ (calculated)), and percent deviation.

#	pIC_50_ (observed)	pIC_50_ (calculated)	Residue	Deviation (%)
**2a**	4.37	4.49	− 0.12	2.75
**2b**	4.45	4.45	0.00	0.00
**2c**	4.53	4.41	0.12	2.65
**2d**	4.57	4.43	0.14	3.06
**2e**	4.60	4.72	− 0.12	2.61
**2f**	4.56	4.41	0.15	3.29
**2g**	4.61	4.70	− 0.09	1.95
**2h**	4.57	4.44	0.13	2.84
**3a**	5.60	5.46	0.14	2.50
**3b**	5.54	5.39	0.15	2.71
**3c**	5.80	5.78	0.02	0.34
**3d**	5.59	5.47	0.12	2.15
**3e**	5.85	5.77	0.08	1.37
**3f**	5.66	5.69	− 0.03	0.53
**3i**	6.70	6.61	0.09	1.34
4a	5.28	5.16	0.12	2.27
**4b**	*4.52*	*4.76*	− *0.24*	*5.31*
**4c**	5.05	4.89	0.16	3.17

The HOMO and LUMO energies are important properties in chemical and pharmacological processes because these properties give information on the electron-donating and electron-accepting character of a compound. It is possible to notice that the E_HOMO_ for the most active studied compounds are the more negative ones (Table 2). This means that the more active compounds are not so good electron-donor molecules when compared to the less active ones.

The PSA is a molecular descriptor extensively used to characterizing the transport properties of drugs, related to its intestinal absorption and the penetration of the blood–brain barrier. According to the model (Eq. 18), together with E_HOMO,_ it is a key descriptor to explain the biological activity of the quinoxaline derivatives.

These descriptors shows that not only steric but also electronic properties are important to understand the interaction between quinoxaline derivatives that present antileishmanial activity and the biological receptor. The steric properties are related to the positioning of the molecule when interacting with the receptor, while the electronic properties are related to the intensity of the molecular association due to electronic interaction.

Among the 18 compounds, only one (**4b**) presents a deviation greater than 5% from the experimental activity value, characterizing it as an outlier. Compound **4b** has bulky substituents, altering its physicochemical properties (such as a much larger area and volume values—see Table 2) when compared to the other compounds of the series, and consequently making discrepant the relationship between structure and biological activity through the proposed equation.

**Figure 4:**
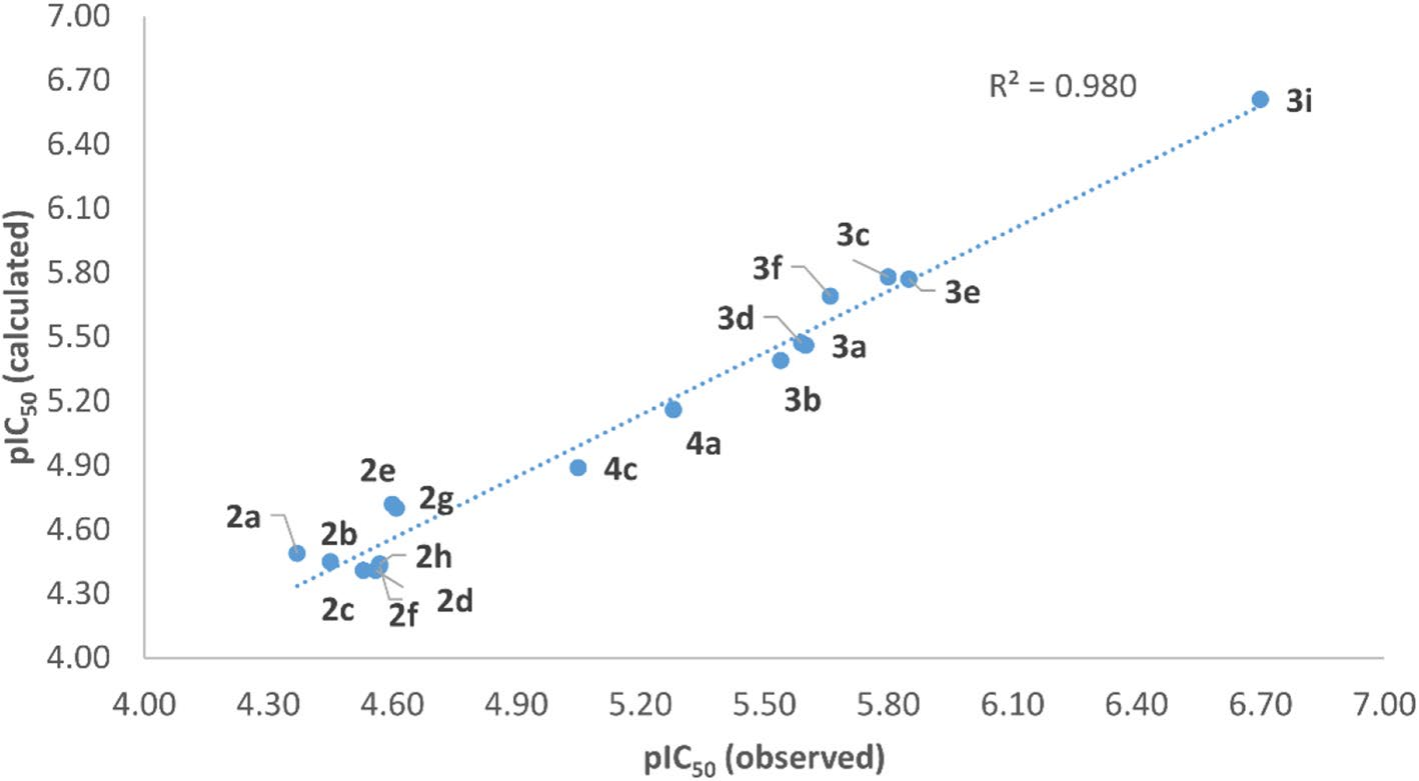
Experimentally observed antileishmanial activity values (pIC_50_ (observed)) *versus* calculated activity values (pIC_50_ (calculated)), using Eq. 1, for the 17 quinoxaline derivatives in the training set (after removing outlier **4b**).

Excluding the outlier 4b, coefficients were recalculated for Eq. 18 providing Eq. 1 (N = 17, R^2^ = 0.980, R^2^_Adj_ = 0.977, s = 0.103, and R^2^ from the leave-one-out-cross-validation (Q^2^) = 0.971), in which analysis of residues (Table 7) and plot of pIC_50_ (calculated) versus pIC_50_ (observed) (Fig. 4) did not show any outlier.

1 $${\text{pIC}}_{{{5}0}} = \, {-}{ 1}.{51 }{-} \, 0.{96 }\left( {{\text{E}}_{{{\text{HOMO}}}} } \right) \, + \, 0.0{2 }\left( {{\text{PSA}}} \right)$$

**Table 7:** Observed (experimental) and calculated (Eq. 1) pIC_50_ (M) values, residuals (pIC_50_ (observed)–pIC_50_ (calculated)), and percent deviation after removing outlier **4b**.

#	pIC_50_ (observed)	pIC_50_ (calculated)	Residue	Deviation (%)
**2a**	4.37	4.49	− 0.12	2.75
**2b**	4.45	4.45	0.00	0.00
**2c**	4.53	4.41	0.12	2.65
**2d**	4.57	4.43	0.14	3.06
**2e**	4.60	4.72	− 0.12	2.61
**2f**	4.56	4.41	0.15	3.29
**2g**	4.61	4.70	− 0.09	1.95
**2h**	4.57	4.44	0.13	2.84
**3a**	5.60	5.46	0.14	2.50
**3b**	5.54	5.39	0.15	2.71
**3c**	5.80	5.78	0.02	0.34
**3d**	5.59	5.47	0.12	2.15
**3e**	5.85	5.77	0.08	1.37
**3f**	5.66	5.69	− 0.03	0.53
**3i**	6.70	6.61	0.09	1.34
**4a**	5.28	5.16	0.12	2.27
**4c**	5.05	4.89	0.16	3.17

Since literature data indicates that there is evidence that only models validated externally, after internal validation, can be considered reliable and applicable for external prediction and regulatory purposes^22,23^, the model was applied for external molecules.

Carrying out an external validation, it was possible to confirm the robustness of the proposed model (Eq. 1). A set of four compounds (**2i**, **3g**, **3h**, and **4d**) was used as external test, representing about 20% of the quantity of observations (N = 22). The test group with its values of observed and calculated pIC_50_, residuals, and percentual deviation are shown in Table 8, where is possible to verify that all of them present a deviation smaller than or equal to 5% of the biological activity value observed experimentally.

**Table 8:** Observed (experimental) and calculated (Eq. 1) pIC_50_ values, residues (pIC_50_ (observed)–pIC_50_ (calculated)), and percent deviation for the test set compounds.

#	pIC_50_ (observed)	pIC_50_ (calculated)	Residue	Deviation (%)
**2i**	4.52	4.47	0.05	1.11
**3g**	5.54	5.63	− 0.09	1.62
**3h**	6.10	6.32	− 0.22	3.61
**4d**	4.89	4.79	0.10	2.04

### Synthesis of the new derivative and activity prediction by the QSAR model

Unpublished compound 5 was synthesized, characterized by NMR, and its biological activity in the promastigote form of *Leishmania amazonensis* was evaluated. The built and validated QSAR model, corresponding to Eq. 1, was used to predict the activity of this new derivative.

Therefore, the descriptors present in Eq. 1 were calculated for the new compound 5 (E_HOMO_ = − 6.52 eV and PSA = 53.19 Å^2^) and a value of 5.81 was predicted for biological activity (pIC_50_) against *Leishmania amazonensis.* Comparison with the experimental result (IC_50_ = 2.0 ± 1.2 μM and pIC_50_ = 5.70) shows that the QSAR model (Eq. 1) proposed here, presented a good predictive capacity with a deviation of 1.93%, being useful to drive the synthesis of new quinoxaline derivatives, saving time and resources that would be spent on synthesis and testing of biological activity.

### NMR data

7-chloro-*N*-methyl-3-(methylsulfonyl)quinoxalin-2-amine (5).

61% yield. ^1^H NMR (300 MHz, CDCl_3_) δ: 7.77 (d, *J* = 8.9 Hz, 1H), 7.72 (d, *J* = 2.3 Hz, 1H), 7.35 (dd, *J* = 8.9, 2.3 Hz, 1H), 6.93 (m, 1H), 3.41 (s, 3H), 3.12 (d, *J* = 4.8 Hz, 3H). ^13^C NMR (300 MHz, CDCl_3_) δ: 148.68, 144.21, 141.19, 138.69, 132.96, 130.47, 126.38, 125.43, 40.45, 27.92.

## Conclusions

SAR studies of a series of quinoxaline derivatives were carried out and a new quinoxaline derivative was proposed as a potential antileishmanial agent. The unpublished compound was synthesized and tested against *Leishmania amazonensis* promastigotes. A new QSAR model was built, and it was capable to predict the activity of the new compound being useful to drive the synthesis of other ones.

## Data Availability

The authors confirm all data generated and analyzed during this study are available in the article.

## References

[1] World Health Organization. Global report on neglected tropical diseases 2023; Retrieved Aug 2023 from WHO/CED/PHE/18.10; World Health Organization: Geneva, Switzerland, 2023.

[2] Fonseca BP, Albuquerque PC, Zicker F (2020). Neglected tropical diseases in Brazil: Lack of correlation between disease burden, research funding and output. Trop. Med. Int. Health.

[3] World Health Organization. Ending the neglect to attain the Sustainable Development Goals: A road map for neglected tropical diseases 2021–2030; World Health Organization, 2021. Retrieved Feb 2023.

[4] Varikuti S (2018). Host-directed drug therapies for neglected tropical diseases caused by protozoan parasites. Front. Microbiol..

[5] World Health Organization. Leishmaniasis; World Health Organization, 2023. Retrieved Feb 2023.

[6] Tuon FF, Amato VS, Zequinao T, Cruz JAW (2022). Emerging computational technologies in human leishmaniasis: Where are we?. Trans. R. Soc. Trop. Med. Hyg..

[7] Kerru N, Gummidi L, Maddila S, Gangu KK, Jonnalagadda SB (2020). A review on recent advances in nitrogen-containing molecules and their biological applications. Molecules.

[8] Rivera G (2022). Quinoxaline 1,4-di-*N*-oxide derivatives: Are they unselective or selective inhibitors?. Mini-Rev. Med. Chem..

[9] Montana M, Montero V, Khoumeri O, Vanelle P (2020). Quinoxaline derivatives as antiviral agents: A systematic review. Molecules.

[10] Aakasha VB, Ramalakshmia N, Bhuvaneswaria S, Sankaria E, Arunkumarb S (2022). Comprehensive review on versatile pharmacology of quinoxaline derivative. Russ. J. Bioorg. Chem..

[11] Cogo J, Kaplum V, Sangi DP, Nakamura-Ueda T, Corrêa AG, Nakamura CV (2015). Synthesis and biological evaluation of novel 2,3-disubstituted quinoxaline derivatives as antileishmanial and antitrypanosomal agents. Eur. J. Med. Chem..

[12] Cogo J (2018). Quinoxaline derivatives as potential antitrypanosomal and antileishmanial agentes. Bioorg. Med. Chem..

[13] Tropsha A (2010). Best practices for QSAR model development, validation, and exploitation. Mol. Inform..

[14] Cherkasov A (2014). QSAR modeling: Where have you been? Where are you going to?. J. Med. Chem..

[15] SPARTAN’10, Wavefunction Inc. Irvine, CA 92612, U.S.A., 2011.

[16] Sander, T. OSIRIS Property Explorer. Organic Chemistry Portal (2001). https://www.organic-chemistry.org/prog/peo

[17] Karelson M, Lobanov VS, Katrizky AR (1996). Quantum-chemical descriptors in QSAR/QSPR studies. Chem. Rev..

[18] Lipinski CA (2004). Lead- and drug-like compounds: The rule-of-five revolution. Drug Discov. Today: Technol..

[19] Veber DF, Johnson SR, Cheng H, Smith BR, Ward KW, Kopple KD (2002). Molecular properties that influence the oral bioavailability of drug candidates. J. Med. Chem..

[20] Hansh C, Unger SH (1973). Strategy in drug design. Cluster analysis as an aid in the selection of substituents. J. Med. Chem..

[21] Gaudio AC, Zonade E (2001). Proposição, validação e análise de modelos que correlacionam estrutura química e atividade biológica. Quím. Nova..

[22] Gramatica P (2007). Principles of QSAR models validation: Internal and external. QSAR Comb. Sci..

[23] Golbraikh A, Tropsha A (2002). Beware of q2!. J. Mol. Graph. Model..

